# Cystic Fibrosis: Overview of the Current Development Trends and Innovative Therapeutic Strategies

**DOI:** 10.3390/pharmaceutics12070616

**Published:** 2020-07-02

**Authors:** Fahad A. Almughem, Ahmad M. Aldossary, Essam A. Tawfik, Mohammad N. Alomary, Waleed S. Alharbi, Mohammad Y. Alshahrani, Abdullah A. Alshehri

**Affiliations:** 1 National Centre for Pharmaceutical Technology, King Abdulaziz City for Science and Technology (KACST), P.O. Box 6086, Riyadh 11442, Saudi Arabia; falmughem@kacst.edu.sa (F.A.A.); etawfik@kacst.edu.sa (E.A.T.); 2 National Centre for Biotechnology, King Abdulaziz City for Science and Technology (KACST), P.O. Box 6086, Riyadh 11442, Saudi Arabia; aaldossary@kacst.edu.sa (A.M.A.); malomary@kacst.edu.sa (M.N.A.); 3 Department of Pharmaceutics, Faculty of Pharmacy, King Abdulaziz University, P.O. Box 80260, Jeddah 21589, Saudi Arabia; wsmalharbi@kau.edu.sa; 4 Department of Clinical Laboratory Sciences, College of Applied Medical Sciences, King Khalid University, P.O. Box 61413, Abha 9088, Saudi Arabia; moyahya@kku.edu.sa

**Keywords:** cystic fibrosis, cystic fibrosis transmembrane conductance regulator (CFTR) gene, gene editing, nanocarriers

## Abstract

Cystic Fibrosis (CF), an autosomal recessive genetic disease, is caused by a mutation in the gene encoding the cystic fibrosis transmembrane conductance regulator (CFTR). This mutation reduces the release of chloride ions (Cl^−^) in epithelial tissues, and hyperactivates the epithelial sodium channels (ENaC) which aid in the absorption of sodium ions (Na^+^). Consequently, the mucus becomes dehydrated and thickened, making it a suitable medium for microbial growth. CF causes several chronic lung complications like thickened mucus, bacterial infection and inflammation, progressive loss of lung function, and ultimately, death. Until recently, the standard of clinical care in CF treatment had focused on preventing and treating the disease complications. In this review, we have summarized the current knowledge on CF pathogenesis and provided an outlook on the current therapeutic approaches relevant to CF (i.e., CFTR modulators and ENaC inhibitors). The enormous potential in targeting bacterial biofilms using antibiofilm peptides, and the innovative therapeutic strategies in using the CRISPR/Cas approach as a gene-editing tool to repair the CFTR mutation have been reviewed. Finally, we have discussed the wide range of drug delivery systems available, particularly non-viral vectors, and the optimal properties of nanocarriers which are essential for successful drug delivery to the lungs.

## 1. Introduction

Cystic fibrosis (CF) is a recessive genetic disease caused by a mutation in the epithelial chloride channel—cystic fibrosis transmembrane conductance regulator (CFTR). CF is a predominant genetic disorder with a disease severity ranging from mild to life-threatening. The number of CF patients in a population varies depending on ethnicity. Previous reports have revealed that one in every 3000 newborns suffer from CF in Northern Europe, whereas this ratio is one in every 10,000 newborns in Latin America [1,2]. The CFTR gene, one of the ATP-binding cassettes (ABC) on chromosome 7, encodes a cyclic AMP (cAMP)-activated chloride channel [3,4]. CFTR is found in different organs like the pancreas, lungs, and sweat glands, and a gene mutation in CFTR results in different types of CF diseases [5]. Numerous diseases like sinusitis, liver disease, and meconium ileus can be seen in CF patients [2]. The CF mutation database enlisted approximately 2076 mutations in the CFTR [6]. However, not all mutations in the CFTR gene cause CF. In fact, according to the clinical and functional translation of CFTR (CFTR2) website, 23 gene variants of CFTR out of the 432 variants identified in 89,052 CF patients, do not cause CF [7]. Mutation in the CFTR gene either results in fewer CFTR channels or has a negative impact on the functioning of these channels. For instance, mutation in CFTR in the pancreas reduces the functioning of CFTR channels, resulting in the defective functioning of the pancreas [8]. As the CFTR channels on the epithelial cells of the pancreatic duct release bicarbonate (HCO_3_^−^) to neutralize the pancreatic fluid and maintain it at its optimum pH [9,10], abnormalities in their function can lead to obstruction of the pancreatic duct [11]. The primary and most common organ affected by CF is the lung. Briefly, in CF, the defective CFTR reduces the release of Cl^−^ ions, thereby hyperactivating the epithelial sodium channels (ENaC) and causing the absorption of more Na^+^ ions. This results in the dehydration and thickening of the formed mucus, making it a good environment for microbial growth [12,13]. Current therapeutic strategies for curing lung CF focus on repairing the CFTR mutation, inhibiting ENaC functions, breaking down the thickened mucus using mucolytic medications, and targeting the inhabitant microbes. This review has first examined the pathogenesis of CF. Subsequently, it has covered the current gene-based and non-gene-based therapeutic approaches relevant to pulmonary complications of CF diseases, particularly focusing on the innovative therapeutic strategies that could be implemented in the near future. Finally, the role of different viral and non-viral nanocarriers in the delivery of therapeutics to the lungs has been evaluated.

## 2. Pathogenesis of Cystic Fibrosis (CF)

### 2.1. Mutation in Cystic Fibrosis Transmembrane Conductance Regulator (CFTR)

The leading cause of CF is a genetic alteration in the CFTR [14,15]. CFTR, an ABC transporter complex, is regulated by the phosphorylation of cAMP and intracellular ATP. It has multiple functions, some of which are still not completely defined [16,17]. It is located on the apical side of the epithelial tissue in various parts of the body, including the lungs, where one of its primary roles is to function as a Cl^−^ channel. It also maintains the transmembrane flow of other electrolytes like HCO_3_^−^, Na^+^, and potassium (K^+^) ions across the cell membrane, which aid in controlling the mucus viscosity of the airway epithelial cells [18,19]. Therefore, a defect in the CFTR gene fundamentally affects the ion transport function of the CFTR protein, which reduces anion secretion and enhances the absorption of ENaC-mediated Na^+^ ions in the airway [20].

Several genetic mutations can lead to the development of CF. Based on De Boeck and Amaral’s classification [21], CFTR mutations are divided into seven classes (I to VII). In class I, nonsense and frameshift mutations, including G542X, R1162X, and W1282X, affect protein production by mediating premature transcription termination, which leads to the development of a protein with either missing amino acids or a new amino acid sequence. These unstable mRNA transcripts are more liable to degradation by a nonsense-mediated decay (NMD) mechanism [22]. Most of the CF mutations belong to class II, including R560T, A561E, R1066C, and N1303K [23,24,25,26], as well as the most common F508del that occurs on at least one allele in 90% of CF patients. Class II mutations affect the structural conformation of the corresponding CFTR protein. These misfolded proteins remain associated with chaperons and are ultimately recognized and subjected to the Endoplasmic Reticulum-Associated Degradation (ERAD) pathway [27].

Typically, phosphorylation of the regulatory domain, ATP binding, and hydrolysis of the nucleotide-binding domains (NBD) controls the activity of the CFTR channel gating. Class III mutations, such as G551D, are located in NBD, and disrupt the regulation of the CFTR channel by restraining ATP binding to these domains, leading to a decline in channel transport activity [28]. Class IV mutations, including R117H, R334W, and R234P, affect the amino acids found in the channel pore, and cause a decrease in the channel’s conductivity [29]. The influence of class V mutations, such as 3272−26A>G, is observed in the amount of normal CFTR protein produced. The protein level is significantly reduced due to a splicing mutation, which results in the generation of insufficient normal transcripts [30]. Class VI mutations, for instance c.120del123, contribute to a reduction in surface retention and the stability of CFTR in the plasma membrane [31,32]. Although the effect of class VII mutation is similar to that of class I since the CFTR protein is not formed, its severity is greater. Here, no mRNA is synthesized, owing to a large deletion in the CFTR, such as dele2,3(21 kb) [33]. Class VII mutations are untreatable, owing to the lack of any corrective therapy [21].

### 2.2. Hyperactivation of Epithelial Sodium Channels (ENaC)

Hydration of the epithelial surfaces of different tissues is crucial for tissue homeostasis and normal physiological functions, which is maintained by airway surface liquid (ASL) in the lungs and regulated by ENaC [34]. In addition to controlling the sodium balance, ENaC aids in the maintenance of blood volume and pressure [35]. Therefore, ENaC dysfunction results in a number of local and systemic diseases like renal salt wasting and CF. The former is caused by the loss of ENaC function [36], whereas the latter is developed due to the improper functioning of ENaC [13,37].

ENaC is a heterotrimer protein channel consisting of α, β and γ subunits, and is located in the apical surface of epithelial cells [38]. The α and γ subunits have extracellular loops, which upon cleavage, activate the ENaC and enhance Na^+^ uptake, while the β-subunit reportedly has a regulatory role [39]. In some tissues, such as the brain, kidneys, and human nasal epithelium, a fourth ENaC subunit (δ) is present. This fourth subunit alters the biophysical features of the ENaC channels; tetrameric αβγδ-ENaC channels generate a two-fold constitutive Na^+^ current larger than that generated by the trimeric ENaC channels [35,40].

ENaC activity is regulated by different intracellular and extracellular factors. At the cellular level, the expression of ENaC subunits is under the control of several factors like TGFβ, serine/threonine kinase SGK1, proteases, and aldosterone [41]. Following its assembly, ENaC is activated via proteolytic cleavage by channel activating protease 1. Several other cellular cascades, like the ERK pathway, ligase protein Nedd4-2, cAMP, PIP2, and purinergic signaling, are implicated in ENaC regulation [42,43]. Additionally, external factors affecting the expression and activity of ENaC include ions, proteolytic cleavage, and mechanical stress [44].

In the lung airway, regulation of ENaC function is carried out by the CFTR protein, which controls the absorption of Na^+^ ions by inhibiting ENaC function [45]. In addition to its regulatory activity, CFTR has an absorptive capacity and works with or against ENaC [46]. Therefore, *CFTR* mutation is associated with the depletion of Cl^−^ ions and concomitant enhancement in the absorption of Na^+^ ions. CFTR-independent activation of ENaC has also been reported. For instance, the release of the elastase enzyme by neutrophils and the cleavage of cathepsin B activates the ENaC subunit [47]. During infection, enzymes like protease and alkaline protease that are released by some bacteria can also activate ENaC and elevate Na^+^ ion absorption [48]. Moreover, mutation in the genes encoding α and β subunits induce CF-like illness in the absence of CFTR mutations [49]. In the lungs, the presence of ASL and the consequent mucociliary clearance is mediated by the regulation of Cl^−^ ion secretion by CFTR and Na^+^ ion absorption by ENaC [34]. The hyperactivation of ENaC generates an osmotic gradient and finally leads to the dehydration of ASL. The consequent accumulation of hyperviscous mucus and impairment of mucociliary movement represent the hallmarks of CF [12,50].

**Figure 1:**
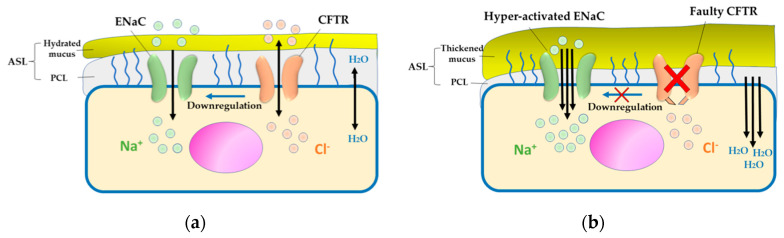
Pathogenesis of cystic fibrosis (CF). (**a**) In normal epithelial cells, salt and water secretion is coordinated by cystic fibrosis transmembrane conductance regulator (CFTR) and absorption via ENaC, which hydrates the airway surface liquid (ASL). (**b**) In CF airway epithelium, defective CFTR impairs Cl^−^ ion and water exchange, causing epithelial sodium channels (ENaC) hyperactivation and leading to dehydrated ASL and thickened mucus, which is considered a conducive environment for infection and inflammation.

In contrast to ENaC hyperactivity, mutations leading to ENaC hypoactivity have been reported to cause pseudohypoaldosteronism. This disease is characterized by elevated airway secretions and extremely rapid mucus removal [49,51]. Although other channels contribute to the hydration of the airway, a balance between CFTR and ENaC is important for the maintenance of homeostasis in the ASL [52]. However, the effect of CFTR on ENaC activity is debatable [53]. Some studies support the notion that ENaC action is abnormal and relies on the CFTR in CF patients [54,55,56], while other studies have reported that ENaC activity is unaltered in both CF and normal individuals [57,58]. Figure 1 summarizes the pathogenesis of CF, correlating CFTR and ENaC.

### 2.3. Complications Associated with Mutation of CFTR and Hyperactivation of ENaC

The major complications of CF include lung destruction and respiratory failure [53]. Owing to a mutation in the CFTR encoding the chloride channel found in the apical membrane of several epithelial cells, including the lungs, the level of apical Cl^−^ ion reduces, while ENaC activity increases [12,59,60,61]. This leads to an imbalance between the Cl^-^ ion secretion and Na^+^ ion absorption through CFTR and ENaC, respectively. This imbalance depletes the volume of ASL, which is necessary for the clearance of respiratory mucus [59,62], thereby leading to the formation of thickened dehydrated mucus which serves as a nidus for bacterial infections [60,63]. In addition, the normal mechanism of mucus clearance depends on the movement of cilia which are located in the apical surface of the epithelial cells inside the periciliary layer (PCL) and their heads across the mucus layer. In normal conditions, cilia can move easily because of the watery layer of PCL that will lead to the removal of the trapped materials in the mucus layer toward the mouth [64]. In CF patients, cilia movement is inhibited due to the thinning of the PCL and the thickened mucus layer, which cannot repel the inhabitant materials, such as microorganisms [64]. This mucus then acts as a barrier for the delivery of drugs either into the mucus layer itself or across it to the airway epithelial cells [62]. Additionally, attainment of homeostasis of the imbalanced fluid and ions leads to the activation of inflammatory responses, primarily the NLRP3 inflammasome [65]. Thus, the accumulation of the thickened mucus inside the airways and the difficulty in its clearance, as well as the chronic bacterial infection and inflammation could contribute to the destruction of the lung and the respiratory failure in CF patients [62].

One bacterial strain, *Pseudomonas aeruginosa (P. aeruginosa)*, complicates the treatment strategy in CF patients, owing to its ability to colonize new sites and to form biofilms using the quorum sensing system [66,67]. Hence, the bacterium protects itself from the host immune system and antibiotics [68,69]. Even if an antibiotic penetrates the biofilm, owing to its ability to survive in low oxygen environments and use aerobic, as well as, anaerobic metabolic pathways (i.e., aerobe to facultative anaerobe), it is tolerant to most antibiotics (antibiotic resistance), thus making it difficult to eradicate [69,70,71]. Microorganisms like *Staphylococcus aureus (S. aureus)* and other oropharyngeal bacterial flora have proven to be responsible for CF lung infection, either alone or by co-infection with *P. aeruginosa*, resulting in a polymicrobial lung infection [67]. CFTR mutation leads to the presence of thick, dry, and sticky mucus which promotes infection due to enhanced bacterial adherence and lack of bacterial clearance [68]. Therefore, it is necessary to develop a novel drug delivery system which can penetrate the biofilm and existent mucus to eradicate the bacteria. It is also important to elucidate the mechanism of biofilm formation, and its components, in order to develop a suitable therapeutic strategy.

#### 2.3.1. Biofilms in CF

**Figure 2:**
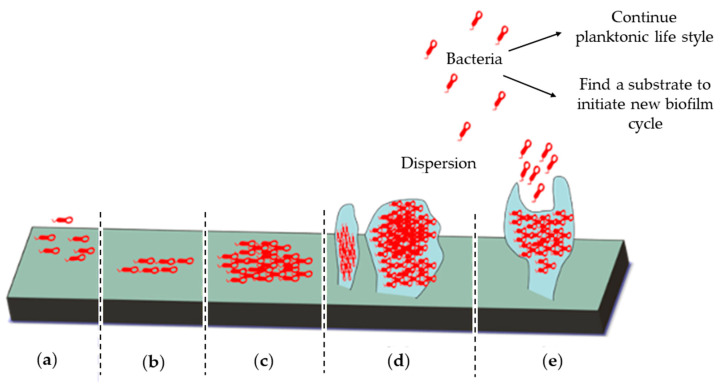
The five stages of biofilm formation. (**a**) Irreversible attachment of bacteria to a substrate, (**b**) bacterial proliferation, (**c**) shift from the bacterial planktonic to the biofilm life style by the loss of bacterial motility and production of extracellular polymeric substances (EPS), (**d**) biofilm maturation, and (**e**) dispersion of bacteria either to colonize a new substrate or shift to their original planktonic life style.

The chronic inflammation and inadequate eradication of pulmonary infections are significant issues in CF patients [72]. These patients suffer from frequent and persistent lung infections caused by ESKAPE pathogens (*Enterococcus faecium, S. aureus, Klebsiella pneumoniae, Acinetobacter baumannii, P. aeruginosa, and Enterobacter species*); most commonly is *P. aeruginosa*, as previously mentioned [73,74]. The ability of some of these organisms to form biofilms results in antibiotic resistance and the development of an innate immune response [75]. Biofilms are organized, adherent bacterial colonies, encapsulated in extracellular polymeric substances (EPS) made of carbohydrates, proteins, and nucleic acids [76]. EPS preserves the integrity of the biofilm and protects it from extreme environmental conditions [77]. As shown in Figure 2, biofilms are formed through five sequential stages [78].

Antimicrobial resistance (AMR) is one of the most common challenges that can hinder an effective treatment of CF. AMP can be naturally occurring in some microorganisms due to their unique structural or functional characterization. For instance, the natural feature of the Gram-negative bacteria, such as *P. aeruginosa*, can resist the glycopeptide antibiotics, for example vancomycin, which is effective against the Gram-positive bacterial strains [79]. Moreover, biofilm-forming bacteria are more AMR than non-adherent planktonic bacteria [80]. Biofilm may not only act as a barrier for antibiotics, but also can facilitate the bacterium to acquire AMR [81]. Biofilms could also enhance the horizontal gene transfer between bacteria, which will cause a transfer of several drug resistance plasmids and thus, sharing of a broad range of resistance genes [80].

The drug efflux system is considered as one of the main strategies that bacteria use to develop AMR. These pumps are mainly encoded by genes presented either in the bacterial chromosome or in plasmid [82]. Drug efflux pumps in bacteria can be categorized into five different classes—the adenosine triphosphate (ATP)-binding cassette (ABC) superfamily, the resistance-nodulation-division family (RND), the major facilitator superfamily (MFS), the small multidrug resistance (SMR) family, and the multidrug and toxic compound extrusion (MATE) family [83]. When the bacterial drug efflux pumps are upregulated, the antibiotics will be expelled from their cellular targets, and therefore, reduce their effectiveness [84]. Thus, targeting these pumps using efflux pump modulators can be considered as a potential strategy to overcome AMR.

In *P. aeruginosa*, the overexpression of four efflux pumps of the RND family, MexAB-OprM, MexCD-OprJ, MexEF-OprN, and MexXY/OprM, plays a major role in enhancing bacterial resistance toward antibiotics [85]. One of the modulators that might be used as an alternative therapy to treat mild infections are essential oils from aromatic plants [84]. For example, cinnamaldehyde (CAN), cinnamon bark essential oil, showed a significant inhibitory activity in vitro against *P. aeruginosa*. This is due to the interaction of aldehyde group of CAN with multiple bacterial cellular components and functions, including membrane lipids, ATPases, and cell division [85]. Furthermore, CAN may also interfere with the quorum sensing regulatory systems of *P. aeruginosa*, which would reduce the chances of biofilm formation [85].

#### 2.3.2. Biofilm Components

The proteins implicated in *P. aeruginosa* biofilms include lectin A (LecA), LecB, and Cyclic Dimeric Guanosine Monophosphate Regulated Two-Partner Secretion A (CdrA) [86]. *P. aeruginosa* CdrA has the ability to bind to carbohydrates, which promote the interaction between matrix molecules [87]. CdrA interacts with the *P. aeruginosa* polysaccharide synthesis locus (Psl), making it a key factor in biofilm formation [88]. In liquid cultures, CdrA overproduction enhances cell auto-aggregation [89]. A *CdrA* mutant exhibited a reduced biofilm biomass and structural integrity, suggesting that CdrA–Psl interaction could facilitate the crosstalk between Psl and biofilm components [90]. Furthermore, numerous studies have reported the importance of extracellular DNA (eDNA) as a structural component of biofilms. For instance, the deoxyribonuclease (DNase) enzyme isolated from *Bacillus licheniformis* acts as an antibiofilm agent in marine bacteria [91]. It has also been reported that the DNase enzyme induced biofilm dispersal in several *P. aeruginosa* isolated from chronic rhinosinusitis patients [92]. However, biofilms containing large amounts of eDNA are not dispersed by DNase, although, the treatment weakens the biofilm structure [93]. Treatment of biofilms with DNase increases their antibacterial sensitivity [94]. In contrast, the addition of exogenous DNA to *P. aeruginosa* biofilms can increase their resistance to antibiotics like gentamicin and other aminoglycoside antibiotics [95]. These studies support the critical role of eDNA in stabilizing bacterial biofilms. Moreover, Gloag et al. (2013) showed that eDNA maintained an efficient flow of bacterial cells, thereby facilitating cell migration and ensuring sufficient supply of cells [96].

EPS maintains the integrity of biofilms by making it resistant to antibiotics and the host immune system. It acts as a structural platform and facilitates cell–cell interactions and cell–surface attachment [97]. In addition to proteins and eDNA, *P. aeruginosa* produces three important polysaccharides—alginate, Psl, and Pectate lyase (Pel) [98]. During chronic infection, *P. aeruginosa* undergoes a phenotypic change due to a mutation in the *mucA* gene. This leads to the overproduction of alginate, which protects the bacterium from the host immune cells and antimicrobial agents [99]. Psl is composed of mannose, glucose, and rhamnose in a 3:1:1 ratio [100]. Inactivation of the *pslA* and *pslB* genes in *P. aeruginosa* PAO1 strain in vitro alters cell–cell and cell–surface interactions, which maintain biofilm integrity and cell aggregation [101]. It has been shown that Psl elevates c-di-GMP levels leading to the generation of a positive feedback loop resulting in the continuous stimulation of biofilm formation [102]. Therefore, targeting Psl using specific antibodies might serve as a novel approach for protecting against *P. aeruginosa* infection.

## 3. Non-Gene Therapy for CF

### 3.1. Small Molecules

Although the conventional therapy currently in use for CF is primarily based on controlling disease symptoms without treating the underlying cause, this approach might reduce disease progression and enhance the patient’s quality of life [103]. The recent discovery of CFTR modulators has provided a breakthrough in CF treatment, since they can be used to improve or potentially correct the CFTR function and other related complications [104]. Rapid developments in the field of drug discovery, owing to high-throughput screening technologies, have led to the screening of the activity of thousands of small molecules simultaneously, in a very short span of time, thus, aiding the identification of molecules that enhance the function of defective CFTR proteins [105]. Several small molecules that control CFTR malfunction are currently used in the clinical field and many are under investigation.

**Figure 3:**
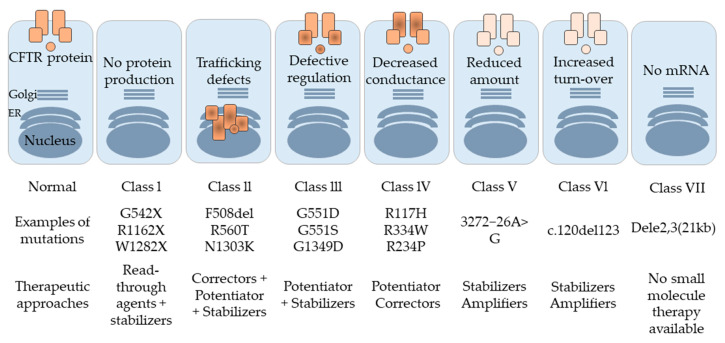
Different classes of CFTR mutations along with the possible therapeutic approach for each class.

Targeting CFTR mRNA or protein via modulators is one of the potential strategies for improving CF therapy by correcting gene malfunction, as shown in Figure 3. Typically, there are four main types of modulators—potentiator, stabilizer, corrector, and amplifier. The potentiator modulator targets the CFTR protein on cell membranes, causing the opening of CFTR ion channels and improving CFTR function [106]. VX-770 (Ivacaftor) is a commonly used potentiator in CF patients. It is the first FDA-approved CFTR potentiator drug and is used especially in cases of G551D class III mutation, which produces a defect in CFTR channel gating. It helps increase the movement of ions across the cell membrane and improves the opening of such defective channels [107]. Several potentiators like QBW251 and PTI-808 are in the process of undergoing clinical trials [108]. Stabilizers, like N91115 (Cavosonstat), are another class of modulators that have been investigated to improve CFTR protein stability. They act by reducing protein degradation in the endoplasmic reticulum (ER) and increasing protein residence time on the cell membrane [109]. The third type of CFTR small molecule modulator is the corrector. This modulator can correct the shape of misfolded CFTR protein, thereby restoring it to its original three-dimensional shape. Thus, it facilitates the functioning of the CFTR protein through its movement toward the cell surface [110]. VX-809 (Lumacaftor) is a corrector used to rectify the F508del mutation, the most common type of CF mutation. This helps avoid the ER-mediated degradation of the CFTR protein by enhancing the interaction between the NBD1, MSD1, and MSD2 domains [111]. Corrector modulators are usually used in combination with potentiator modulators in CF treatment. Examples of this combination include the FDA-approved Orkambi^®^ (lumacaftor combined with ivacaftor) and Symdeko™ (tezacaftor combined with ivacaftor) [112]. Post treatment with Orkambi^®^, a significant in vitro effect has been reported on the transport of Cl^−^ ions, compared to treatment with lumacaftor alone [112]. In the United States, a triple combination of ivacaftor, tezacaftor, and elexacaftor (Trikafta™) has been recently approved for the treatment of CF patients aged 12 years and older carrying at least one F508del mutation [112]. Clinical trial results have demonstrated that this combination increases the CF progression marker, i.e., the predicted percentage of forced expiratory volume in one second (ppFEV1) [113]. The fourth type of modulator is the amplifier. PTI-428, an amplifier, enhances the amount of CFTR mRNA and protein loaded on to the ER. The amplifier is usually combined with a corrector or potentiator modulator [114].

These modulators play a significant role in the treatment of most classes of CFTR mutations except class I, owing to a premature termination codon (PTC) mutation that leads to defective protein production [115]. To overcome this problem, a combination of an aminoglycoside antibiotic like gentamicin or tobramycin, and PTC124 (Ataluren) is used. This serves as an effective treatment regimen since it brings about the insertion of the correct amino acid on the CFTR protein [114].

Alternative therapeutic strategies currently being explored for CF therapy include the use of a combination of CFTR modulators and ENaC inhibitors or calcium-activated chloride channel (CaCCs) agents. Therefore, duramycin and denufosol have been reported as potential CF medicines owing to their ability to enhance the release of intracellular calcium (Ca^2+^) ions [111]. Amiloride, the first ENaC inhibitor to be used in CF therapy, blocks Na^+^ ion channels. However, its use is currently limited, owing to its short half-life and rapid clearance from the body [116]. Although second generation derivatives of amiloride, like benzamil and phenamil, were developed, these also suffer from rapid clearance [117]. Therefore, third generation amiloride derivatives, such as GS-9411, have been developed; however, these demonstrate a potent potassium-sparing ENaC inhibitor effect, which contributes to hyperkalemia [118].

Owing to the low stability and common side effects of amiloride and its derivatives, researchers have been prompted to develop alternative therapeutic strategies that indirectly inhibit ENaC activity, for instance, inhibition of Channel-Activating Protease (CAP) [119]. Camostat, a potent CAP inhibitor, plays an important role in stopping the cleavage of the extracellular loops of ENaC, thus, reducing the transportation of Na^+^ ions across the lung epithelial cells. Despite the significant effect of CAP inhibitors, some concerns like unexpected side effects and lack of efficacy, still exist [50].

SPX-101 (developed by Spyryx Biosciences, Inc. (Durham, NC, USA)), a novel ENaC inhibitor, marks the first nebulized formulation for use in CF therapy to reach clinical trials [120]. It is an inhaled small peptide which mimics the functions of the ENaC modulator, short palate, lung, and nasal epithelial clone 1 (SPLUNC1). It reduces the amount of ENaC on the plasma membrane, thereby reducing Na^+^ ion uptake and ASL absorption. It has been reported that the SPLUNC1 modulator is inhibited in the acidic ASL environment and degraded by the action of proteases found in the CF patients [120]. However, SPX-101 serves as a promising mutation-agonistic ENaC modulator owing to its protease resistance and high stability at low pH [120]. Table 1 summarizes the small molecule therapies in CF and their targeted mutations.

### 3.2. Targeting Bacterial Biofilms

*P. aeruginosa* infection is a well-established cause of morbidity in CF [121], although other pathogens have also been found in the CF lung that could induce biofilm formation. Lung function failure in CF has also been associated with Methicillin-resistant *Staphylococcus aureus* (MRSA) [122]. In addition, *Haemophilus influenzae* frequently colonizes the CF lungs [123], and *Aspergillus fumigatus* has been isolated from sputum of CF patients [124]. CF patients show high levels of *Burkholderia spp* in the salivary fluid [125]. *Streptococcus pneumoniae*, and *Klebsiella pneumoniae* have also been found in CF patients’ sputum [125]. *P. aeruginosa* chronic infection is prevalent in about 80% of adult CF patients [126].

Biofilms have been identified in the CF lung. Biofilm growth in CF is associated with several mutations, adaptation of the bacteria to the lung conditions and resistance to antibiotics [126]. *P. aeruginosa* CF isolates display altered expression of virulence factors which affects motility, antimicrobial resistance, lipopolysaccharide (LPS) structure, and production of some secreted products such as pyocyanin [127]. *P. aeruginosa* pyocyanin might inhibit the function of antioxidants and block Cl^−^ transport in human bronchial epithelial cells [128]. *P. aeruginosa* can also adopt a mucoid phenotype in the CF lung [129]. Once *P. aeruginosa* becomes mucoid and forms biofilm, the eradication of infection is greatly difficult. This leads to immune response disturbance, impaired pulmonary function, and persistence of chronic disease [129].

Antimicrobial peptides (AMPs), which consist of 12 to 50 amino acid residues, are active agents with antimicrobial and antibiofilm activities [130]. They play an essential role in innate immunity in humans as well as other species [131]. For instance, magainins are standard natural AMPs found in the African frog *Xenopus laevis*. Cathelicidins and defensins are important AMPs isolated from human leukocytes, which disrupt the microbial cell wall, and consequently, contribute to innate immune defense [132]. Several studies have shown that AMPs display bactericidal effects, modulate inflammatory responses, and prevent biofilm formation [133,134]. The innate defense regulator-1018 (IDR-1018) peptide is considered to be a promising antimicrobial agent, owing to its effectiveness against *P. aeruginosa*. The antibiofilm activity of AMPs functions via different mechanisms of action [132]. Under stressful conditions, bacteria produce guanosine tetraphosphate and guanosine pentaphosphate [(p)ppGpp], which are essential regulators of biofilm homeostasis [135]. The IDR-1018 peptide directly attaches to (p)ppGpp and thus, affects biofilm formation [136]. However, bacterial biofilms are typically embedded in EPS, which protects them from harsh environments and antimicrobial agents [137]. DNase I and glycoside hydrolase dispersin B peptides cause inactivation of eDNAs and cleavage of primary EPS components, respectively, and thus, have potent activities against dispersed bacterial biofilms [72]. Moreover, certain bacteria synthesize amyloids, which promote surface adhesion, aggregation, and biofilm formation [138]. Translocation-dependent antimicrobial spore component (TasA), a significant biofilm component, forms amyloid-like fibers that help in biofilm formation [139]. This amyloid formation can be inhibited by AA-861 and parthenolide AMPs, leading to the attenuation of bacterial virulence and biofilm destruction [139]. As pyocyanin produced by the bacteria promotes the continuous generation of eDNAs that are essential for biofilm formation [140], another antibiofilm strategy is to crosslink these compounds with EPS273 AMP, thereby inhibiting the release of eDNAs [141]. In this review, we have described the antimicrobial action of some AMPs. However, further investigations are required to circumvent the challenges associated with these AMPs. Thus, this review facilitates the development of novel AMPs, thereby providing new opportunities in the fields of drug discovery and antibiofilm therapy.

## 4. Gene Therapy for CF

### 4.1. Targeting CFTR

As previous mentioned, the conventional treatment for CF is based on the reduction in disease symptoms and improvement in the patient’s quality of life using antibiotics, physiotherapy, and mucus clearance medications. However, these therapeutic approaches do not target the gene mutation causing the disease.

The cloning of the CFTR gene in 1989 opened doors for the correction of CF at the genetic level [4,142,143]. Theoretically, the use of gene therapy for CF is more advantageous compared to other genetic diseases. As CF is caused by a single gene mutation, CF carriers are phenotypically normal. The airway is easily accessible for drug delivery through a pulmonary nebulizer, and a relatively low level of CFTR correction has a high therapeutic potential (6–10% of normal CFTR activity is needed to restore the epithelial cells electrical properties and 25% to restore mucociliary function) [144,145].

In the last three decades, numerous efforts have been made to develop a gene replacement therapy for faulty CFTR with either cDNA or mRNA molecules by using viral or non-viral vectors. The earliest clinical study in this field was conducted in 1993, wherein CFTR cDNA was administrated through an adenovirus (AV) vector, in which the early-transcribed region E_1_ was deleted to prevent viral replication. AV has been an attractive vector for CF gene therapy, owing to its efficiency in transducing the non-dividing cells of the airway without genome integration. However, the low transfection efficiency and immune inflammation response against the virus, even with low doses, makes this therapeutic approach infeasible for CF patients [146].

The mRNA approach involves translation of mRNA molecules in the cytoplasm of the transfected cells without the need for translocation into the nucleus, which makes them more effective compared to DNA-based therapy. Moreover, the risk of potential insertion into the host genome is less compared to that with DNA molecules. The main limitations of this approach are the short half-life, expression instability, and potential cytotoxicity through toll-like receptor activation [147]. Chemical modification, using various techniques, has showed great improvements in the mRNA-based approach, especially in cancer therapy and vaccination for infectious diseases [148]. In vitro studies on differentiated human nasal epithelial primary cells revealed that the minimal dose of wt-CFTR-mRNA (0.6 μg/cm^2^) delivered by lipofectamine 2000 unregulated the functional electrophysiology of the CFTR channel for over 72 h [149]. The efficacy of this approach was also demonstrated in vivo by Robinson et al. (2018), where the CFTR mRNA restored the Cl^-^ ions efflux by 55% in CFTR knockout mice compared to healthy mice [150]. Moreover, in 2018, the first clinical trial for mRNA therapeutic phase I/II (NCT03375047), sponsored by Translate Bio, Inc. (Lexington, MA, USA), was given to 40 adult participants regardless of the mutation class. Eluforsen (QR-010), developed by ProQR Therapeutics, is a 33mer antisense oligonucleotide (wildtype template) that can bind to CFTR mRNA sequences adjacent to the F508del homozygous mutation, and correct it. The clinical trial phase Ib (NCT02564354) for this drug has been completed, where it was found to be well tolerated and showed improvements in the CF Questionnaire-Revised respiratory domain [151]. Thus, the correction of CFTR mRNA using oligonucleotide molecules may restore channel function. Furthermore, antisense oligonucleotide molecules have been investigated to correct splice CFTR mutations (class V) such as c.2657+5G>A [152].

To date, numerous clinical trials have been conducted to determine the ability of gene therapy to reduce the progression of CF lung disease. However, a clinically effective treatment is yet to be established. The crucial challenges in gene therapy for CF include the limited levels of gene transfer achieved within the airway epithelial cells and the persistent expression of the transgene [153].

In recent years, gene editing has been suggested as a potential treatment strategy for several genetic diseases, including CF. This therapeutic approach has a permanent effect and permits intervention at the chromosomal level of a specific gene, leading to the correction of all disease-associated mutations. CFTR function can be rescued by either editing the endogenous gene or inserting a wild type CFTR at a safe harbor locus such as *AAVS1*. The three most prominent gene editing techniques that have been explored as potential CF treatment approaches are zinc finger nucleases (ZFNs), transcription activator-like effector nucleases (TALENs), and Clustered regularly interspaced palindromic repeats (CRISPR)/CRISPR-associated systems (Cas).

**Figure 4:**
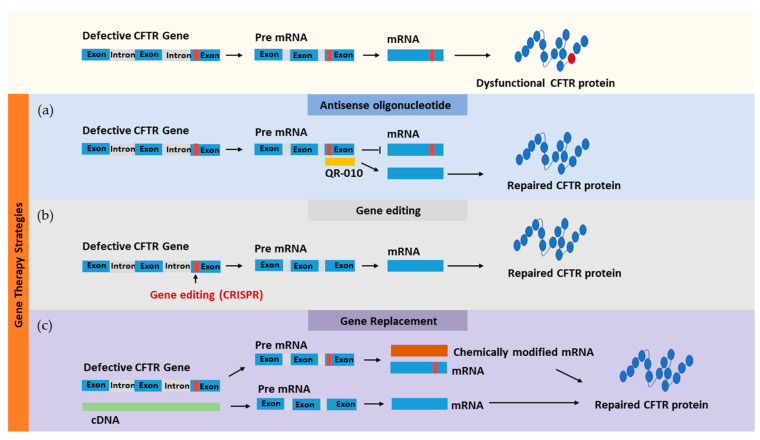
Gene-based therapeutic approaches for targeting faulty CFTR using different methods: (**a**) antisense oligonucleotide, (**b**) gene editing, and (**c**) gene replacement.

ZFN-based gene editing successfully corrected the F508del mutation in CFTR29 cells; however, the editing efficiency was very low [154]. Using a similar approach, induced pluripotent stem cells (iPSC) from CF patients were corrected and differentiated into airway-like epithelial cells by the TALEN gene editing technique, resulting in restoration of their electrophysiological property when co-cultured with CFBE41o- cells [155]. However, since both techniques are laborious, an alternative approach, the CRISPR/Cas technique, has been proposed. This technique is simple and has a low synthesis cost [156,157]. Furthermore, a variant of the CRISPR/Cas system that is capable of precise correction without a mutagenic double-strand break is now available [158,159]. Geurts et al. (2020) demonstrated the ability of base editor systems to recover the genetic mutation and functions of CF intestinal organoids [160]. Consequently, the CRISPR/Cas gene editing system may serve as a potential tool for CF treatment in the near future. Figure 4 summarizes gene-based therapeutic approaches for targeting faulty CFTR.

### 4.2. Targeting ENaC

Since the CFTR gene mutation causes hyperactivation of ENaC, the absorption of Na^+^ ions is enhanced and the lung airway mucus becomes dehydrated. A previous report revealed that the in vivo overexpression of ENaC in mice lungs resulted in increased absorption of Na^+^ ions and reduction in ASL [161]. Therefore, the inhibition of ENaC expression is a promising therapeutic approach for the treatment of CF. One method for the inhibiting the expression of ENaC-encoding genes (*SCNN1A*, *SCNN1B*, *SCNN1G*, and *SCNN1D* encoding α, β, γ, and δ ENaC subunits, respectively) involves the use of a single strand nucleic acid known as antisense oligonucleotide (ASO). When this oligonucleotide is hybridized to mRNA, RNase H is triggered to slice the hybridized mRNA [162]. Targeting the α-subunit of ENaC in the lung, using ASO, could inhibit the cationic channel activity [163,164,165]. Another study showed the ability of aerosolized ENaC ASO containing wing modifications to inhibit ENaC mRNA in CF-like mice models [148]. This aerosolized ENaC ASO helped cure CF symptoms, like airway hyper-responsiveness and inflammation, and has reached the phase I clinical trial [165].

Another strategy to inhibit ENaC expression is using small interference RNA (siRNA). siRNA is a double stranded RNA, of approximately 21 base pair (bp) RNA nucleotides, consisting of a passenger strand that is identical to a part of the targeted mRNA and a guided strand that is complement to a part of the target mRNA in an open reading frame. When siRNA is taken up by a cell, it removes the passenger strand during the activation of the RNA-induced silencing complex (RISC) [166]. The guided strand is led by the RISC, which contains four Argonaute subunits (Ago 1–4), to its target mRNA, where it is cleaved by Ago 2 [166,167,168]. The primary advantage of gene expression inhibition using siRNA is that the guided strand, presented in RISC, is protected from cytoplasmic endonucleases [169,170]. Additionally, the RISC-incorporated guided strand triggers the cleavage of multiple mRNA targets expressing the same protein [171]. siRNA has also been used to evaluate the genes responsible for the regulation of ENaC expression; the inhibition of diacylglycerol kinase iota protein, which is involved in the phosphatidylinositol biphosphate metabolism, downregulates ENaC function, leading to the normal absorption of Na^+^ ions and fluid in CF airways [172].

The direct inhibition of ENaC by siRNA has been evaluated both in vitro and in vivo [34,173,174,175]. In the in vitro evaluation, the levels of mRNA expressing the α, β, and γ subunits of ENaC were measured in untreated primary bronchial epithelial cells. The results showed that the α subunit was more abundant compared to β and γ subunits [175]. Following siRNA treatment, a reduction was observed in the absorption of Na^+^ ions and fluid, which persisted for over seven days [158]. Another in vitro study showed that a significant increase in ASL was observed only when both the α and β subunits of ENaC were knocked down simultaneously using siRNA [34], while the siRNA-mediated knockdown of the α subunit mRNA alone reduced the function of ENaC [174].

**Figure 5:**
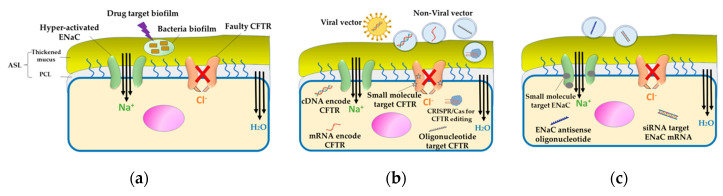
Therapeutic approaches for CF. (**a**) Targeting bacterial biofilm: Bacterial infection in CF is associated with the formation of a biofilm that resists the host immune system as well as antibiotics. Antibiofilm therapy is essential to prevent bacterial colonization and adherence. (**b**) Direct therapy for CFTR: Faulty CFTR is corrected by gene therapy, wherein CFTR is delivered as cDNA or mRNA using a viral or non-viral vector. Another approach is the use of antisense oligonucleotide molecules to repair the mRNA encoded CFTR. Small molecule modulators, like potentiator, stabilizer, corrector, and amplifier are considered as promising agents for the restoration of CFTR function. (**c**) Indirect therapy targeting the hyperactivated ENaC: In CF, owing to the loss of CFTR regulation, the sodium channel (ENaC) is hyperactivated, leading to the dehydration of the mucus layer. Inhibiting ENaC expression using siRNA, antisense oligonucleotide molecules, or small molecule modulators can correct the mucociliary defect.

In vivo knockdown of the α subunit was successfully performed using a cationic liposome carrier containing a peptide ligand [173]. Additionally, it was observed that repeated in vivo doses of this siRNA-liposome lipoplex improved the knockdown of the α subunit of ENaC mRNA [176]. Furthermore, the in vitro translocation of this formulation across the mucus membrane demonstrated its ability to cross the mucus layer faster than the naked siRNA [176]. Therefore, successful gene therapy for CF treatment requires a suitable nanocarrier that can easily penetrate the mucus layer and be taken up by the epithelial cells. Figure 5 summarizes the therapeutic approaches for CF.

## 5. Nanocarriers for CF Treatment

For several years, different therapeutic strategies have been identified to improve the efficiency of CF treatment. Various gene-based and non-gene-based therapeutics have proven to be promising approaches for the treatment of CF [176,177,178,179]. Despite the enormous developments in the management and treatment of CF in recent years, several challenges still exist. One of the primary challenges is the successful delivery of these therapeutics to the target organ. Gene-based therapeutics face the problem of low stability in body fluids since they are degraded by nucleases [180]. Moreover, the off-target effect limits the in vivo application of gene-based therapeutics [177]. In contrast, non-gene-based therapeutics have limitations such as rapid clearance from the body, low stability, and high toxicity during the administration of a large dose. Significant progress in the field of nanotechnology has led to the discovery of nanocarriers, which will improve the current therapeutic strategies for CF treatment and open doors for the development of innovative approaches. Nanocarriers offer a novel strategy to enhance the delivery of therapeutics for CF patients. The implementation of nanotechnology in designing therapeutic vehicles primarily improves the specificity for the drug target cells, thereby reducing unexpected side effects.

Inhalation formulations have been used extensively in CF patients for the local delivery of therapeutics (i.e., antibiotics) to the lungs, evading obstacles like hepatic and renal clearance that are associated with the parenteral administration of these medications [181]. Numerous studies have focused on the promising features of nanoparticles as carriers for the delivery of gene- and non-gene-based molecules via inhalation, to enhance the treatment efficiency and reduce toxicity [182,183]. The field of nanotechnology has overcome the complexity of the pulmonary system with its different biological and physiological barriers, including respiratory tract, epithelial layers, mucus, and bacterial biofilm [184]. However, the biocompatibility and biodegradability of materials used for the production of inhalable nanoparticles must be considered before developing novel inhaled products for CF treatment [185].

One of the barriers for CF therapy is the thick mucus layer present within the alveolar region that prevents the penetration of the nanoparticles to the target cells [67]. The mucus layer consists of different components like mucin fibers, DNA, and actin, which form a crosslinked network of mesh size 100–200 nm in CF patients and 500 nm in healthy individuals [186]. Nanoparticles should be small enough to avoid steric inhibition by the dense fiber mesh and also to avoid adhesion to mucin fibers in order to penetrate mucus successfully [187]. The application of nanoparticles of less than 100 nm enhances the penetration of therapeutic molecules through the mucus layer [187,188]. However, in the late stages of CF, the viscoelasticity increases, resulting in a decrease in the nanoparticles penetration across the mucus layer [187].

The negatively charged components of the mucus layer act as an electrostatic barrier for cationic nanoparticle penetration. Hence, a nanoparticle with a neutral surface-charge (zeta potential values ranging from −10 to +10 mV) will reduce the interaction between the nanoparticles and mucus [112,189]. It is hypothesized that coating particles with a high density of low molecular weight polyethylene glycol (PEG) may reduce adhesive interactions between nanoparticles and mucus. This low molecular weight of PEG could be enough to not entangled with the mucin fibers and that the PEG density is sufficient to effectively shield the hydrophobic core of the nanoparticle and enables their penetration through the mucus layer [190]. Thus, the penetration of nanoparticles through the mucus layer can be enhanced by reducing the electrostatic interaction via conjugation with low molecular weight PEG, which possesses muco-inert properties [191]. Such coating is widely studied as a mucus penetrating approach, and it was recently reported that nanoparticles coated with low molecular weight PEG could rapidly cross the physiological human mucus even with a particle size of 500 nm. Moreover, surface modifications of nanoparticles with a high density of low molecular weight PEG (of 2–5 kDa) reduced the interaction between particles and mucus [192]. Furthermore, decreasing the mucosal viscoelastic property using mucolytic agents, like N-acetylcysteine (NAC) and DNase, is an effective strategy to increase the diffusion of nanoparticles through the mucus. NAC decreases the mucus viscosity by disrupting the disulfide bonds connecting the mucin proteins, whereas DNase breaks down the DNA in the sputum [112].

Local delivery of nanoparticles to the lungs via inhalation or nebulization improves therapeutic efficacy by increasing the dose delivered to the lungs (i.e., localizing) of the CF patient. However, nanoparticles encounter the complex pulmonary system, which acts as a barrier, and confront difficulties in the deposition of the administrated drug. Therefore, inhaled nanoparticles must be designed with the ability to get deposited in the deepest part of the lungs and reach the alveoli upon penetration of the mucus layer. The deposition of an optimal level of inhalable particles in the alveolar region can be achieved by using a formulation of size less than 3 µm [193]. Additionally, the superficial characteristics of nanoparticles and their morphology are also important parameters that affect the deposition of the nanoparticles.

Aerosolization of suspended nanoparticles using nebulizers aids in obtaining a droplet with an appropriate size for delivery into the lungs of CF patients. Owing to the limitations of nebulized nanoparticles like short-term stability and patient compliance, an alternative form of pulmonary dosage called the dry powder inhaler (DPI) was suggested for the delivery of nanoparticle-based formulations to the lungs [194]. This can be achieved by the spray drying of nano-embedded microparticles (NEM) known as Trojan particles, which facilitate the deposition of nanoparticles into the lungs, following their release from the microparticle matrix [194]. NEM powders are formulated by mixing cryoprotectant excipients like mannitol or lactose with poly lactic-co-glycolic acid (PLGA), chitosan, and polyacrylate, which are biodegradable and biocompatible polymers [195].

To improve the treatment efficiency in CF patients, several viral and non-viral vectors have been examined both in vitro and in vivo. Viral vectors like AV, adeno-associated virus (AAV), herpes simplex virus, and retrovirus have been engineered for application in gene therapy. The first clinical trial on the application of viral vectors in CF treatment began in 1993 using an E_1_-deficient AV capable of expressing the CFTR protein in airway epithelial cells. This strategy was used to correct the defective chloride transport in CF patients [146]. Subsequently, more viral vectors like AAV emerged in CF treatment. AAV showed a high transduction efficiency compared to AV. It has been reported that the delivery of an integrated AAV-based CFTR expression vector in a CF animal model stabilized the expression of CFTR and corrected the defects in the anion transport system [196]. Despite the use of viral vectors for the delivery of therapeutics, several limitations have been identified. The high immunogenicity, low loading efficiency, and production complications of viral vectors have promoted researchers to find a safer and more efficient drug delivery system [197].

Non-viral vectors like liposomes, solid lipid nanoparticles (SLNs), polymers, and dendrimers have been commonly used for gene and drug delivery in CF patients. The crucial features of non-viral vectors are their low immunogenicity, easy fabrication, low production cost, and their ability to load both non-gene and gene-based therapeutics.

Liposomes are one of the most commonly used non-viral delivery systems that are exploited as nanocarriers in CF treatment. Liposomes consist of a lipid bilayer with hydrophilic heads and hydrophobic tails. The hydrophilic heads are orientated toward the aqueous core, a bulky environment. Different drugs, antibiotics, peptides, and nucleic acids are entrapped or complexed with liposomes and have been tested as inhalation delivery systems in CF treatment. Local administration improves the therapeutic efficacy of the loaded drugs [197]. Their biocompatibility and ability to encapsulate hydrophilic and lipophilic compounds are the primary advantages of liposomes. The encapsulation of drugs by liposomes increases their bioavailability and reduces cellular toxicity by decreasing the required therapeutic dose. Plasmid DNA (pDNA), siRNA, and other therapeutic molecules used in gene therapy suffer from low stability and difficulty in crossing the cell membrane to reach the site of action. These aspects could be overcome using liposomes. A phase IIb clinical trial (NCT00789867) has reported that the complexing of pDNA (pGM169) with the cationic liposome, GL67A, enabled successful drug delivery in CF patients with enhanced FEV1, using commercially available nebulizers [198]. Using cationic liposomes for the delivery of antibiotics or antibiofilm peptides enhances the electrostatic interaction with biofilm-forming bacteria, allowing longer drug residence time with the pathogen. These key features of liposomes have opened doors for the design of novel therapeutic strategies to develop an efficient non-viral delivery system targeting CF. The transfections of primary CF epithelial cells with cationic liposomes composed of 1,2-di-O-octadecenyl-3-trimethylammonium propane (DOTMA) and 1,2-dioleoyl-sn-glycero-3-phosphoethanolamine (DOPE) complexed with siRNA of α and β-subunit of ENaC has successfully corrected the mucociliary defects while increasing the ASL depth [176]. In another study, targeting of α-subunit of ENaC in CF patients, using a cationic liposomal–siRNA complex conjugated with the target peptide (hydrodynamic size 192  ±  4 nm and ζ potential 23.6  ±  0.4 mV), has modulated the Na^+^ ion hyperabsorption, thereby, restoring the mucus hydration and correcting the mucociliary defects [173]. Two amikacin (an aminoglycoside antibiotic)-loaded liposomal formulations, ARIKACE™ and ARIKAYCE™, that are composed of dipalmitoyl phosphatidylcholine (DPPC) lipid and cholesterol have successfully reached the clinical trials [199,200]. ARIKAYCE™ has reached phase IIa clinical trial (NCT03905642) after evaluating 49 participants, whereas ARIKACE™ has reached phase III clinical trial (NCT01316276) after evaluating 206 participants. Both liposomal formulations have shown better diffusion across the mucus layer than the free drug and were able to deposit the loaded antibiotic in the targeted *P. aeruginosa* biofilm. Moreover, the treatment of chronic *P. aeruginosa* infection in CF patients with inhaled amikacin loaded into neutral liposomes composed of DPPC and cholesterol, has improved the antibiotic penetration of the biofilm, suggesting its potential to reach the infected sites in the lungs [201]. Similarly, the encapsulation of tobramycin into negatively charged liposomes composed of DPPC, 1,2-dioleoyl-sn-glycero-3-phosphocholine (DOPC), and 1,2-dipalmitoyl-sn-glycero-3-phosphoglycerol sodium salt (DPPG), has demonstrated higher antibiofilm efficiency on *Burkholderia cepacia* complex (Bcc) infection in CF patients compared to the free drug [202]. In spite of the advantages of using liposomes as local delivery systems for CF treatment, the nebulization process, which affects the stability of the liposomes, remains a major concern. However, preparing liposomal formulations as DPI, using lyophilization or spray drying technology, has been reported to have a significant improvement on the stability of the liposome [203].

SLN, a lipophilic crystalline matrix, is another non-viral delivery system that could be used in CF therapy. The high physiochemical stability, ease of scaling up, and low cytotoxicity are the key advantages of SLNs [182]. However, the encapsulation capacity is lower than that of liposomes. A recent study has reported the successful delivery of chemically modified CFTR mRNA using SLNs that is (hydrodynamic size of 104.2 ± 30.5 nm) composed of dilinoleylmethyl-4-dimethylaminobutyrate (DMA), distearoylphosphatidylcholine (DSPC), cholesterol, and 1,2-dimyristoyl-rac-glycerol (DMG) [150]. The study findings indicated that the Cl^-^ ion secretion was restored and the CFTR channel was opened [150].

Polymeric nanoparticles function as important drug carriers in CF treatment. Different biocompatible and biodegradable polymers have been widely investigated for use as non-viral delivery systems. Additionally, polymeric nanoparticles have several advantages like high stability and the ability to modify their surfaces by adding certain functional groups or target ligands [204]. Promising results were obtained when PLGA nanoparticles loaded with ciprofloxacin (hydrodynamic size 190.4 ± 28.6 nm) and used on different strains of *P. aeruginosa* to improve the antibacterial activity, reduce the required dose, and enhance the penetration across the mucus layer, thus, enhancing the efficiency of CF antimicrobial therapy [205]. In gene therapy, the injection of polymeric nanoparticles (chitosan-coated PLGA) loaded with chemically modified human CFTR mRNA into the lungs of CFTR-deficient mice, resulted in a significant decrease in Cl^−^ ion secretion and improvement in the critical lung function parameters [206]. Using poloxamine-based copolymer with tetrafunctional structure could be a potential non-viral vector for lung gene therapy. In a recent study, Guan et al. (2019) developed nanoparticles composed of poloxamine 704 and synthetic peptides that self-assembled with nucleic acid molecules. The peptide–poloxamine nanoparticles mediated high transfection of mRNA and pDNA in vitro as well as in the lungs of CF mice without exhibiting cytotoxicity [207]. Additionally, the FDA-approved drug PS-341, a proteasomal pathway inhibitor, loaded in PEGylated PLGA nanoparticles (hydrodynamic size 121.5 ± 15 nm) showed controlled and sustained drug release [208].

Dendrimers are synthetic, nanosized, highly branched molecules structurally composed of three main regions—the core, dendritic branches, and functional head groups [209]. The most commonly used dendrimers are the polyamidoamine (PAMAM) dendrimers family [185]. Dendrimers have several advantages over the frequently used nanocarriers. They possess low polydispersity index and can easily perform chemical modifications, thereby allowing better internalization of the loaded drugs across the membrane of the target cells [210]. The beneficial properties of dendrimers have enhanced their use as nanoplatforms for antimicrobial therapy and gene delivery [210]. Dendrimers can be formulated as DPIs or suspensions for administration via inhalation in CF patients [211]. As previously mentioned, *P. aeruginosa* infection, which is known to form biofilms and possess antimicrobial resistance, is considered as a serious complication in CF [212]. The cysteamine drug (Lynovex^®^, Novabiotics), an oral mucoactive, antibiofilm, and antibacterial agent, has passed the phase IIb clinical trial (NCT03000348) for use in the treatment of acute, infectious CF exacerbations [213]. PAMAM dendrimers were modified as cysteamine-like structure dendrimers (PAMAM-DEN^CYS^), to obtain the antibiofilm activity of cysteamine [214]. PAMAM-DEN^CYS^ demonstrated enhanced penetration across the thick mucus layer, thus, reducing biofilm formation, and the infection associated with *P. aeruginosa* [214]. A recent study by Faraj et al. (2019) has confirmed the novel activity of PAMAM-DEN^CYS^ in inhibiting *P. aeruginosa* infection, and its role in the progression of CF therapy with respect to the recovery of F508del-CFTR in the plasma membrane [215].

Exosomes have attracted attention as novel drug delivery systems owing to their unique composition [216]. They are nanosized, extracellular vesicles (EVs) with mean diameters ranging from 50 to 200 nm, and properties similar to that of unilamellar (single-layered) liposomes [217]. Exosomes are secreted from different cell types and exhibit novel properties like specific cell targeting ability and homing selectivity, which primarily depends on the cell from which it originated [218]. The lipid bilayer of exosomes is composed of different types of phospholipids (i.e., glycosphingolipid ganglioside GM3, phosphatidylserine, phosphatidylethanolamine and phosphatidylcholine). This structure protects the loaded drugs from the opsonin protein and other immune components. Hence, it reduces the interaction with blood components and mediates the delivery of biomolecules and genetic materials to the target cells [219]. The surface of exosomes contains several essential proteins and ligands like integrins and tetraspanins, which have an intrinsic function of inducing the cellular internalization of therapeutics to the target sites [220,221]. A recent study has demonstrated the treatment of human nasal epithelial cells of F508del CF patients with the CRISPR/dCas–VPR and BGas–gapmer systems (BGas is a long, non-coding RNA involved in transcriptionally modulating CFTR expression with a gapmer) via the activation of endogenous CFTR. This gapmer was loaded inside exosomes isolated from A549 cells and lipid-based nanoparticles. The study findings revealed enhanced expression of the CFTR following the application of both nanoparticles. However, the expression was higher in exosomes compared to lipid-based nanoparticles [180]. The composition of exosomes can be therapeutically effective, in which they give a synergistic effect when they are used as nanocarriers. It was reported that the intratracheal administration of EVs, such as exosomes that are originated from mesenchymal stem cells (MSCs), can be an effective treatment for bronchopulmonary dysplasia (BPD) in an animal model [222]. In their study, the number of alveoli in the BPD animal model was significantly increased when these EVs were administrated intratracheally. Another study demonstrated an in vitro dose-dependent efficacy of EVs that are originated from adipose MSCs contain alpha 1 antitrypsin protein, which is known for its role in the lung as an elastase inhibitor [223]. Furthermore, EVs from adipose MSCs contain approximately 46 proteins which have antibacterial activities against Gram-negative bacteria such as *P. aeruginosa* and *Klebsiella* [223].

Despite the significant advantages of using nanocarriers in the delivery of gene-based and non-gene-based therapeutics in CF patients, certain challenges still exist. The elimination of nanoparticles by the immune system and macrophages is considered as the primary barrier for inactivating nanoparticles and increasing their clearance from the body [224]. The local administration of nanoparticles, via inhalation, directly into the lungs, helps reduce this clearance. Another strategy is the coating of nanoparticles with PEG to induce steric hindrance (known as the stealth effect), thereby protecting the nanoparticles from degradation or elimination by the immune system [191]. Nevertheless, the complexity of the respiratory tract makes the development of inhaled nanoparticles a challenging process. The deposition of nanoparticles deep in the lung is another concern for consideration, since the inhaled drugs must penetrate to the mucus layers covering the alveoli. Table 2 summarizes the nanocarriers commonly used for gene and drug delivery in CF patients.

## 6. Conclusions and Future Direction

Our review has explained the pathogenesis of CF and the different treatment approaches that are recommended. Despite the well-identified cause of CF, its treatment strategies have proven to be very challenging. Unfortunately, no model therapeutic strategy exists for CF treatment since the variation in the mutated gene is different from patient to patient. The conventional therapeutic approaches for CF primarily focus on improving CFTR function and its associated complications. This review has covered a wide range of therapeutic approaches for use in CF patients. Small molecule modulators demonstrate significant clinical efficacy by enhancing the pulmonary function in certain CF patients, thus improving their quality of life. However, in other CF patients, where the modulators are not applicable, a combination of different classes of medications, with different mechanisms of action, can be involved in the treatment plan. Targeting bacterial biofilms using antibiofilm peptides, and using gene-editing tools like CRISPR have shown great potential for use in CF therapy. These innovative approaches, when delivered through a nanocarrier system, have proven to be significant in the treatment of CF.

Typically, a proper gene editing therapy for CF involves a clear understanding of the mechanism of this approach, for instance, the amount of the cells needed to correct CFTR function, the type of cells in the airway tissue that will be targeted, and their lifespan. These values need to be investigated in vivo, as the genetic and non-genetic modifiers affect the in vitro testing. Additionally, this in vivo assessment must be extended to involve other channels interacting with the CFTR, like ENaC, to evaluate the restoration of CFTR function needed for clinical benefit. The gene editing technology has improved dramatically since the use of the CRISPR/Cas system in 2013 [156,157]. Nevertheless, more precautions are required while using this system owing to the off-target effect, wherein the CRISPR system can manipulate a similar genetic sequence that occurs in another genome. Another concern that needs to be considered is the immune response to the CRISPR components, gRNA and Cas9, especially in a repeat dosing regimen, which can lead to serious complications.

Moreover, the airway epithelial tissue consists of various types of cells. Cells with predominant gene expression are considered as preferable targets for gene therapy. A recent study has identified a new cell type within the human bronchial epithelial tissue and mouse tracheal epithelial tissue known as the pulmonary ionocyte. This cell exhibits a high expression of CFTR (60%) compared to the ciliated cells (4%) [225,226]. However, further study is required to develop a suitable vector that can efficiently target these cells.

Finally, personalized medicine is considered to be the future of CF therapy. Here, the treatment is customized according to each patient’s genomic identity. Small molecule modulator is a kind of personalized medicine used for a specific mutation class. Additionally, the expression of transforming growth factor beta 1 (*TGF-β1*) and mannose-binding lectin 2 (*MBL2*) have been identified as gene modifiers for CF and infection severity [227]. Other CF modifier genes that have been reported through genome-wide association studies (*GWAS*) are Ets homologous factor (*EHF*) and APAF1 interacting protein (*APIP*) [228].

## Figures and Tables

**Table 1:** Cystic fibrosis (CF) small molecule therapies and their targeted mutations.

Name	Therapeutic Approach	Target CFTR Mutations	Development Stage	Ref
Ivacaftor	Potentiator	Class III (G551D mutation)	FDA-Approved, 2012	[107]
N91115 (Cavosonstat)	Stabilizer	Class II (F508del homozygous)	Phase II/Discontinued	[109]
Orkambi^®^ (lumacaftor + ivacaftor)	Corrector + potentiator	Class II (F508del homozygous)	FDA-Approved, 2015	[112]
Symdeko™ (tezacaftor + ivacaftor)	Corrector + potentiator	Class II (F508del homozygous)	FDA-Approved, 2018	[112]
Trikafta™ (ivacaftor + tezacaftor + elexacaftor)	Potentiator + corrector + corrector	Class II (F508del heterozygous)	FDA-Approved, 2019	[113]
PTI-801/PTI-808 /PTI-428	Corrector + potentiator + amplifier	Class II (F508del homozygous)	Phase II	[108,114]
PTC124 (Ataluren)	Read-through	Class I (PTC mutation)	Phase III/Discontinued	[114]
Duramycin	Cl^−^ stimulator through CaCCs	-	Phase II/Discontinued	[111]
Denufosol	Cl^−^ stimulator through CaCCs	-	Phase III/Discontinued	[111]
SPX-101	ENaC inhibitor	-	Phase II	[120]

**Table 2:** Non-viral vectors commonly used for gene and drug delivery in CF patients.

Nanocarrier	Composition	Drug	Key Finding	Clinical Trial Phase and No.	Ref
Liposomes	GL76A	pGM169	Increase in FEV1 and lung function stabilization	Phase IIb NCT01621867	[198]
	DOTMA/DOPE	siRNA	Efficient restoring of mucus hydration and airway clearance	Preclinical	[176]
	DOTMA/DOPE/targeting peptide	siRNA	Effective correction of mucociliary defects	Preclinical	[173]
	DPPC/Chol	Amikacin	Used for treatment of *P. aeruginosa*	Phase III NCT01316276	[199]
	DPPC/Chol	Amikacin	Liposomal formulation target *P. aeruginosa* biofilm with longer half-life than amikacin alone	Phase IIa NCT03905642	[200]
	DPPC/Chol	Amikacin	Improved penetration within *P. aeruginosa* biofilm	Preclinical	[201]
	DPPC/DOPC/DPPG	Tobramycin	Enhanced antibiofilm effect against *Bcc* bacteria compared to free tobramycin	Preclinical	[202]
SLN	DMA/DSPC/Chol/DMG	cmCFTR	Positive CFTR restoration	Preclinical	[150]
Polymeric nanoparticles	PLGA	Ciprofloxacin	Improved antimicrobial activity and enhanced mucus penetration	Preclinical	[205]
	PLGA/chitosan	cmRNA	Reduced chloride secretion and restoration of lung functions	Preclinical	[206]
	Poloxamine	mRNA/pDNA	Enhanced mRNA and pDNA expression without exhibiting cytotoxicity	Preclinical	[207]
	PLGA/PEG	PS-341	Sustained and more effective drug release and penetration	Preclinical	[208]
Dendrimers	PAMAM-DEN^CYS^	Cysteamine	Inhibition of *P. aeruginosa* growth and restore CFTR function	Preclinical	[215]
Exosomes	A549 isolated	CRISPR/dCas-VPR and BGas-gapmer	Enhanced CFTR expression	Preclinical	[180]
